# Preparation of Bentonite/Chitosan Composite for Bleaching of Deteriorating Transformer Oil

**DOI:** 10.3390/polym12010060

**Published:** 2020-01-01

**Authors:** Hujian Chen, Kewang Zheng, Aohui Zhu, Zhifei Meng, Wei Li, Caiqin Qin

**Affiliations:** 1 School of Chemistry and Materials Science, Hubei Engineering University, Xiaogan 432000, China; 2 Key Laboratory of Biological Resources and Environmental Biotechnology, Wuhan University, Wuhan 430000, China

**Keywords:** chitosan, bentonite, calcination, bleaching, transformer oil

## Abstract

A novel adsorbent containing chitosan (CS) and bentonite (BT) was developed by mixing, drying, and calcining, and used as an adsorbent for the efficient bleaching of deteriorating transformer oil. The effects of calcination temperature, dosage of CS, adsorbent content, adsorption temperature, and adsorption time on the bleaching capacity of transformer oil were investigated. The structure of the adsorbent was also investigated by Fourier-transform infrared spectroscopy (FTIR), X-ray diffraction (XRD), thermogravimetric analysis (TGA), scanning electron microscopy (SEM), energy dispersive X-ray analysis (EDX), transmission electron microscopy (TEM), and N_2_ adsorption-desorption isotherm techniques. The results showed that there was only physical interaction between CS and BT; CS did transform to carbon (C) and covered the surface of BT. The specific surface area and micropore volume of the adsorbent were affected by the calcination process. The composite adsorbent offered an excellent bleaching performance. When the calcination temperature was 300 °C and dosage of CS was 5%, the composite adsorbent had the optimum bleaching properties. When the composite adsorbent content was 4%, the adsorption temperature was 50 °C and the adsorption time was 75 min, the colour number and transmittance of the deteriorating transformer oil decreased from no. 10 to no. 1 and increased from 70.1% to 99.5%, respectively.

## 1. Introduction

Transformer oil is derived from petroleum and plays an important role in transformers, disconnectors, current transformers, and other electrical equipment [1]; however, due to its oxidation in air and thermal decomposition, transformer oil ages and deteriorates, which leads to the production of acids and other oxidation intermediates [2,3,4]. These oxidation products could be further oxidised to form resin, asphalt, and other harmful substances, thus leading to the electrical and physico-chemical performance of the oil decreasing as evinced by deepening of its colour, decreasing breakdown voltage, increasing dielectric dissipation factor and so on [5,6]. It was reported that over 75% of high-voltage transformer failures were attributed to the deterioration of transformer oil [7]. Therefore, the electrical and physico-chemical properties of deteriorated transformer oil could be effectively improved by purification. According to practical experience, the re-use rate of deteriorated transformer oil could reach 90%, which confers important benefits in terms of energy savings and environmental protection. At present, common means of purification involve the use of vacuum oil filters, which could improve the pH, breakdown voltage and other electrical properties of transformer oil, leading to the properties of the recycled transformer oil approaching or reaching those of new oil [8,9]. The colour is one of the important indicators of the quality and condition of transformer oil: it can directly indicate the degree of oil aging. Therefore, it is essential to adsorb pigments, gum or other harmful matters from the deteriorated transformer oil before its re-use, however, traditional adsorbents and vacuum oil filters have little effect on the discoloration of deteriorated transformer oil. Hence, it was both important and desirable to develop a high-efficiency adsorbent for the bleaching of deteriorated transformer oil.

Bentonite (BT) is a natural mineral with a crystal structure composed of layered silicates. Due to the flaky morphology, large specific surface area, moderate cationic exchange capacity, and reactive hydroxyl groups on its surface, the BT has been widely used for the bleaching of edible oil and mineral oil [10]. It was reported that the BT has low oil adsorption, is easily separated, and offers near-perfect decolouring efficiency in edible oil. Activated carbon (C) is also a good adsorbent, and is widely used in the adsorption of various oils, such as edible oil and lubricants [11]. Practice showed that a small amount of activated carbon added to BT decolouriser could enhance the bleaching of edible oil, but, as a simple physical mixture, could only improve the bleaching efficiency to a limited extent. Additionally, the BT would cover the surface of activated carbon and block some of its pores; thus decrease the bleaching efficiency of active carbon and BT.

Chitosan (CS), as a natural polymer of basic polysaccharides, contains large amounts of hydroxyl (–OH) and amino (–NH_2_) groups, which could be easily attached to the surface of CA via the electrostatic interaction between negatively-charged CA and positively-charged CS [12,13,14,15,16]. Some researchers have prepared carbon composite adsorbent materials based on silicates and CS, and used them for the bleaching of edible oils [17,18]. For example, Tian [19] prepared a carbon-attapulgite composite by a calcination method using CS as the carbon source and used it for bleaching of palm oil; the results showed that the bleaching efficiency of the composite was increased over that of pure attapulgite; however, few reports of the bleaching experiment of deteriorated transformer oil through carbon/BT composite are available, hence CS could be used as a carbon source for incorporation within the BT to enhance the bleaching capacity of the adsorbent material. The aim of this work is to develop a novel porous composite based on BT and CS and investigate the influence of calcination temperature, amount of CS, adsorption time, adsorption temperature and the amount of adsorbent on the decolouration properties of deteriorated transformer oil.

## 2. Experimental Work

### 2.1. Material

Natural BT was purchased from Henan Hongshu Environmental Co., Ltd. (Zhengzhou, China), and its chemical composition is: SiO_2_ (50.4%), Al_2_O_3_ (9.6%), MgO (3.1%), Fe_2_O_3_ (3.4%), CaO (4.6%), and K_2_O (1.3%). CS was supplied by Zhejiang Golden-Shell Pharmaceutical Co., Ltd. (Hangzhou, China). The deteriorated mineral transformer oil was supplied by Xiaogan Power Supply Company (Xiaogan, China). Other regents were all supplied by Sinopharm Chemical Reagent Co., Ltd. (Shanghai, China).

### 2.2. Synthesis of Carbon/Bentonite Composite

The C/BT composite could be prepared by calcining the CS/BT composite. Typically, 6% of CS (based on BT) was dispersed in 200 mL 4% (*v*/*v*) hydrochloric acid under constant agitation for 3 h. Then, 15 g of BT was added, and stirred at 700 rpm for 3 h again. Thereafter, the mixed solution was centrifuged at 5000 rpm for 10 min, then the solid matter was dried at 105 °C for 12 h. The CS/BT composite was calcined at 200, 250, 300, 350, and 400 °C for 60 min at a heating rate of 4 °C/min under argon to optimise the calcination temperature. At the optimal calcination temperature at 300 °C, different dosages of CS (1%, 3%, 5%, 7%, and 9%, respectively, based on BT) was added to the BT to obtain an optimised dosage of CS. For comparisons, neat BT and CS were used as the control adsorbent. Information pertaining to the samples is provided in Table 1.

### 2.3. Adsorption Experiments

In the decolouring experiments, 100 mL deteriorated transformer oil was poured into a 250 mL adsorbing container, then different dosages of adsorbent (1%, 2%, 3%, 4%, and 5%, respectively, by mass of oil) was added and stirred at different temperatures for different times to absorb any colouring matter. The transmittance of oil was determined by an UV–VIS spectrophotometer (722A, Jingke, China) with an incident wavelength of 475 nm. The colour number of the oil was recorded using a petroleum colour-meter (SYD-0168, Shanghai, China) by the method of China Standard SH/T0168-92.

### 2.4. Characteristic Analysis

FTIR spectra of the material were obtained by using a Thermo Nicolet iS5 spectrophotometer (Thermo, USA). The material’s crystallinity was recorded by a D8-Advance X-ray diffraction (Bruker, Germany). Measurements were performed at 40 kV and 40 mA current within 10° ≤ 2θ ≤ 80°. The thermal stability was analysed using a TGA (STA449F5, Netzsch, Selb, Germany) within the temperature range of 30–800 °C at a heating rate of 10 °C/min under an N_2_ flow of 40 mL/min. SEM and EDX were performed using a ZEISS sigma300 to analysis the topographic characteristics of the materials. TEM was performed using a JEOL JEM-2100F (Tokyo, Japan) to analyse the internal features. The specific surface area was measured by a Conta ASAP2020 (USA). The adsorption experiments data were processed using SPSS (Ver. 19) software, and expressed as the mean ± SD.

### 2.5. Electrical Properties Analysis

The close flash point of the transformer oil was determined using a flash tester (DZY-002, Dalian Instruments, Dalian, China) by the method of China Standard GB/T261-2008. The breakdown voltage of the oil was assessed using an insulating oil dielectric strength tester (HR-1001, Huarui-broad, Wuhan, China) by the method of China Standard GB/T507-202. The dielectric dissipation factor of the oil was performed through a dielectric loss instrument (MCK6100A, Hubei Meicui Power, Wuhan, China) using the China Standard GB/T5654-2007 codified method.

## 3. Results and Discussion

### 3.1. FTIR Analysis

**Figure 1:**
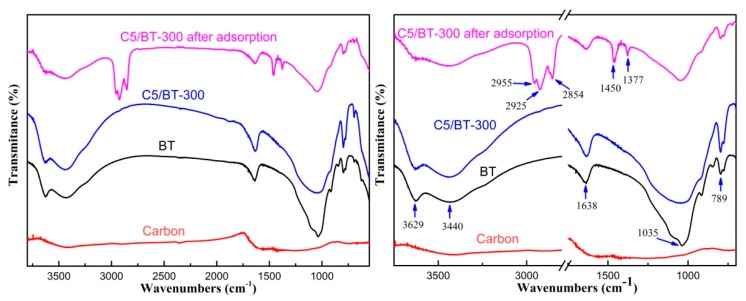
FTIR spectra of different adsorbents.

The FTIR spectra of carbon, BT, C5/BT-300, and C5/BT-300 after adsorption are shown in Figure 1. As can be seen, the carbon showed no peaks. In the spectra of BT, the peak at 3629 cm^−1^ was probably due to the stretching vibrations of Al-OH-Al [20,21]. The band at 3440 cm^−1^ was attributed to the bleaching earth interlayer water molecule stretching vibrations, and the band at 1638 cm^−1^ corresponded to the bending vibration of absorbed and zeolitic water in the channels [22]. The peak at 1035 cm^−1^ was attributed to Si-O stretching, and that at 789 cm^−1^ corresponded to Si-O quartz impurity. When chitosan and BT were mixed, possible chemical interactions could be suggested by changes in characteristic spectra peaks. The spectrum of C5/BT-300 was similar to that of BT, which suggested that chitosan carbon had not destroyed the functional groups and active sites of BT. Additionally, no new peak appeared, and no old peak disappeared, which suggested that there had been no chemical interaction between chitosan carbon and BT: however, some changes were seen in the spectra of C5/BT-300 after adsorption. New peaks at 2955 cm^−1^, 2925 cm^−1^ and 2854 cm^−1^ were assigned to antisymmetric stretching vibration of −CH_3_ and the stretching vibration of −CH_2_. The new peaks at 1450 cm^−1^ and 1377 cm^−1^ were ascribed to the stretching vibration of C = C in the aromatic ring and symmetrical deformation vibration of −CH_3_. Furthermore, the intensity of the main peaks of C5/BT-300 adsorbent decreased significantly after absorption in the transformer oil. These changes were probably due to the presence of colouring matter which was adsorbed by the adsorbent or the small amount of residual oil present.

### 3.2. XRD Analysis

**Figure 2:**
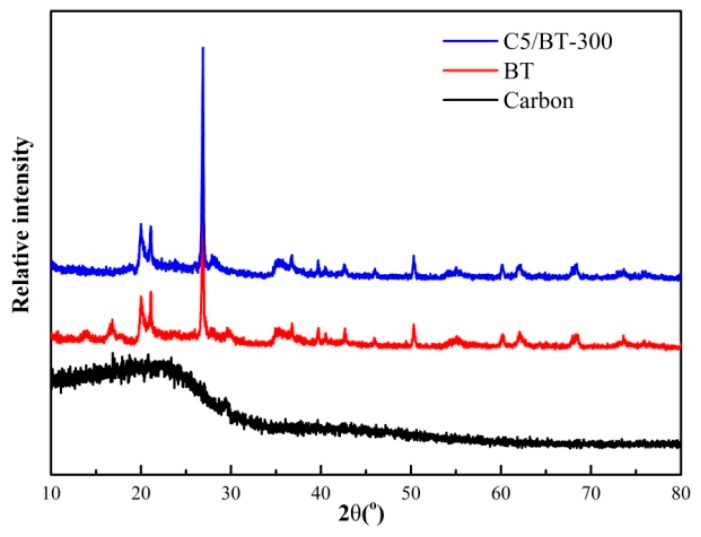
XRD patterns of different adsorbents.

The XRD patterns of carbon, BT and C5/BT-300 are shown in Figure 2 where it can be seen that the chitosan carbon has an amorphous structure, but the neat BT exhibited good crystallisation. When chitosan was added to BT and then calcined at 300 °C, the XRD patterns underwent no significant change, suggesting that the crystalline structure was not destroyed, and the reactions occurred only on the surface of the BT. This also indicates that the calcination temperature was appropriate.

### 3.3. Thermogravimetric Analyses

**Figure 3:**
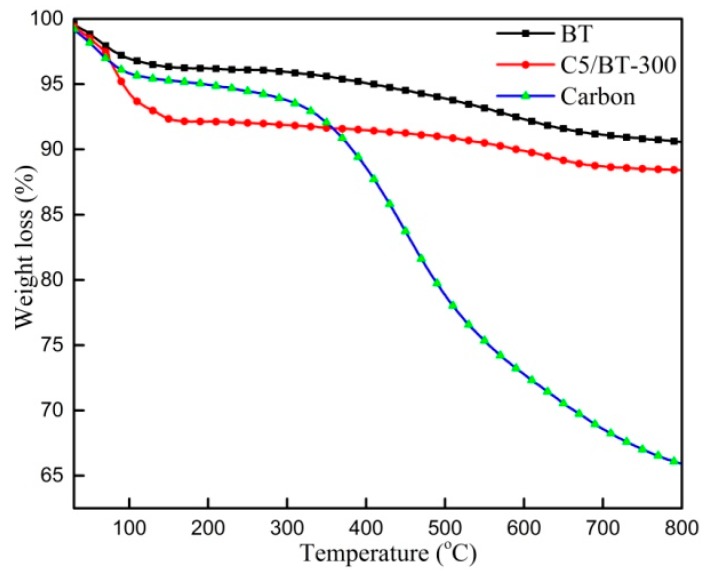
TGA graphs of different adsorbents.

The thermal degradation behaviours of BT, C5/BT-300 and carbon adsorbents are shown in Figure 3. Individual BT presented two stages of weight loss: the first (at around 85 °C) was probably due to the evaporation of residual water from the adsorbent. The second stage (beginning at 330 °C) resulted from the degradation of bound water and zeolitic water in the channels. Likewise, two similar stages in the process of thermal decomposition were found in the C5/BT-300 adsorbent, however, the carbon showed a different thermogravimetric curve: the first stage (at around 80 °C) was associated with the evaporation of residual water; the second stage (beginning at 320 °C) corresponded to the degradation of carbon. The results indicated that the calcination temperature of the composite adsorbent should not exceed 300 °C. The data were supported by those from the adsorption experiment.

### 3.4. SEM and SEM-EDX

**Figure 4:**
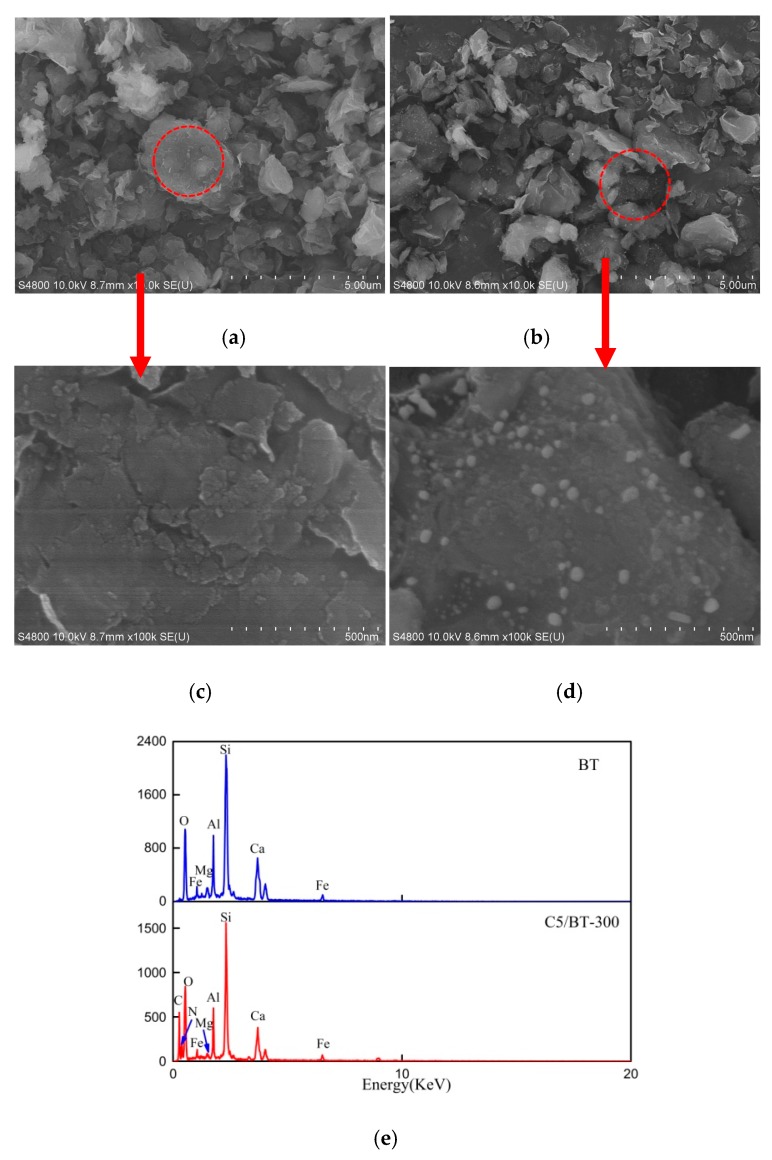
High-resolution SEM images: (**a**,**c**) BT and (**b**,**d**) C5/BT-300. (**e**) EDX of BT and C5/BT-300.

Figure 4 shows the general morphology of BT and C5/BT-300 adsorbent particles. The SEM images in Figure 4a,c show that the BT has an irregular sheet-like structure with a smooth surface, however, different morphologies of C5/BT-300 were observed in Figure 4b. A thin carbon layer covered the surface of the BT, and some carbon particles were distributed roughly on the carbon layer surface, which indicated good bonding between BT and CS. This was probably due to the calcination of CS adhering to the surface of the BT. In addition, the EDX was used to evaluate the elemental composition of BT and C5/BT-300 adsorbents. As can be seen in Figure 4e, the BT contains oxygen, silicon, aluminium, calcium and traces of iron and magnesium, but barely any carbon. Carbon and nitrogen were however present on the surface of the CA as evinced by the EDX analysis, although the amounts thereof were low. This indicated that the CS was transformed to carbon (C) and incorporated into the BT.

### 3.5. TEM Analysis

**Figure 5:**
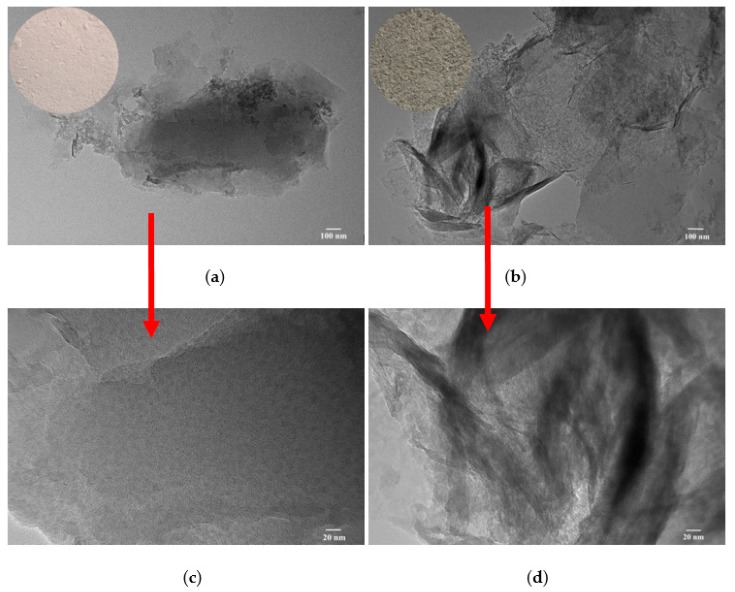
TEM images of (**a**,**c**) BT and (**b**,**d**) C5/BT-300.

Figure 5 shows the TEM images of BT and C5/BT-300 adsorbents. As can be seen, the BT shows a smooth, compact, even structure, without any crumpled features or covering substances; however, when the BT was modified by CS and calcined at 300 °C, the images of C5/BT-300 adsorbent in Figure 5c,d clearly show an uneven morphology, and with some non-homogeneous substance covering the surface of the BT, which can be attributed to the carbon formed by carbonisation of CS. More intuitively, the carbonisation of CS into carbon can be confirmed by the digital photograph of samples before and after modification. The BT shows a light-red, powdered structure, and C5/BT-300 shows the same structure but with as ash-black appearance. This indicated that BT was successfully modified by CS.

### 3.6. BET Analysis

**Figure 6:**
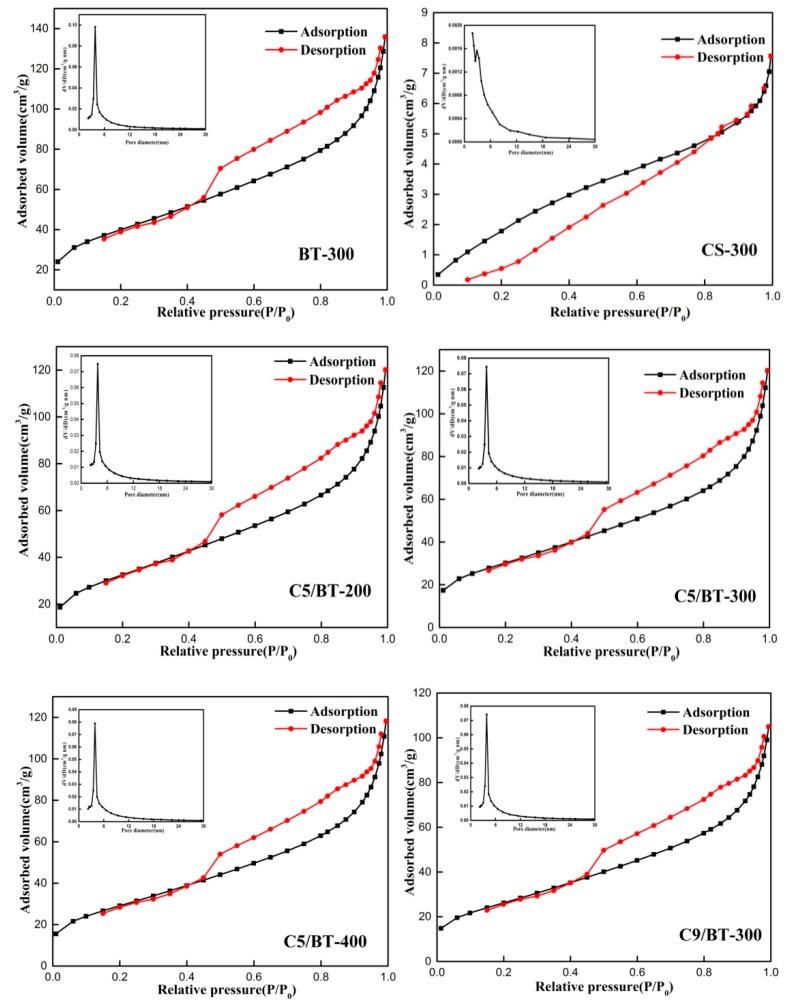
Linear isotherm plots of different adsorbents.

The pore structure parameters of different samples are presented in Table 2 and Figure 6. As can be seen, the specific surface area (*S*_BET_) of BT-300 has a maximum at 141.15 m^2^/g, and gradually decreased with increasing calcination temperature and CS content. For the micropore volume (*V*_micro_*)* of C/BT adsorbents, it slightly increased with increasing calcination temperature but decreased with the addition of CS. These changes were probably due to the following reasons: firstly, the CS covered the surface of the BT, thus leading to the decreased *S*_BET_ and *V*_micro_ of C/BT composite adsorbents. With increasing calcination temperature, CS was gradually transformed into carbon species and size of the carbon granule also decreased, blocking some adsorption channels and decreasing *S*_BET_. With increasing CS content, more organic carbon was formed, which covered the surface of the BT and blocked some channels, thus decreasing *S*_BET_ and *V*_micro_. Secondly, when the calcination temperature was higher, the zeolitic water and bound water of the BT were more likely to be lost, thus increasing the aperture width in the BT. These phenomena were consistent with the results arising from the adsorption experiment.

### 3.7. Optimisation of Adsorbent

#### 3.7.1. Effect of Calcination Temperature

**Figure 7:**
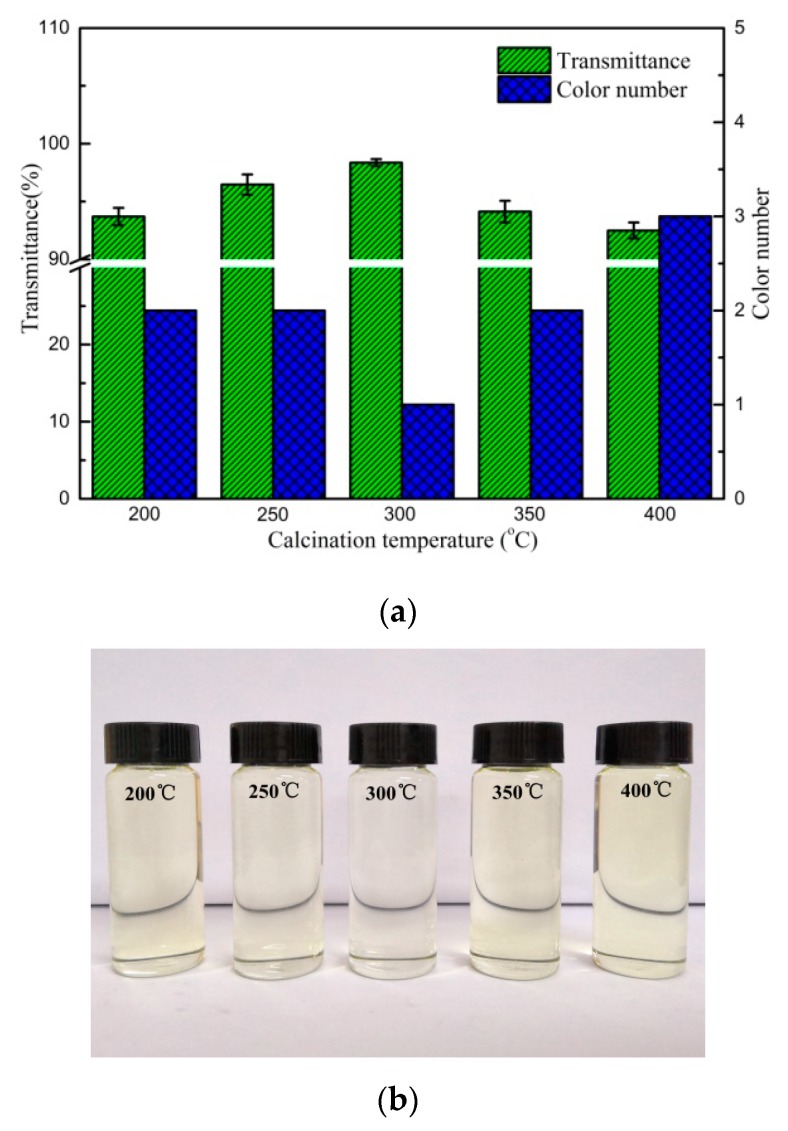
(**a**) Effect of calcination temperature on the transmittance and colour number of the oil (4% of C5/BT adsorbent at 50 °C for 2 h). (**b**) Effect of calcination temperature on the appearance of the oil (4% of C5/BT adsorbent at 50 °C for 2 h).

Figure 7a,b show the effect of calcination temperature on transformer oil bleaching capacity for C5/BT composite adsorbent. It can be found when the calcination temperature was 300 °C, the bleaching capacity of C5/BT-300 adsorbent was superior to that of other adsorbents. It can be seen that the transmittance of the oil increased gradually and the colour number decreased when the calcination temperature was increased from 200 °C (93.7%) to 300 °C (98.4%), but decreased and increased at higher temperatures, respectively. As can be seen from Figure 1, when the calcination temperature was 300 °C, the colour of the oil was lightest. An increase in transmittance and decrease in colour number of the oil could be attributed to the gradually carbonised CS molecules adhering to the surface of BT. When the calcination temperature was lower, the CS molecules could not completely form carbon. With increasing temperature, the carbon could be completely formed, and the pore structure parameters of adsorbents increased, which contributed to the bleaching capacity of the adsorbent for the transformer oil; however, the carbon might be decomposed and the pore parameters could be decreased when the calcination temperature was higher (over 300 °C), which led to the decrease of the bleaching capacity of the adsorbent. Therefore, based on the data set obtained, the optimal calcination temperature was 300 °C.

#### 3.7.2. Effect of CS Dosage

**Figure 8:**
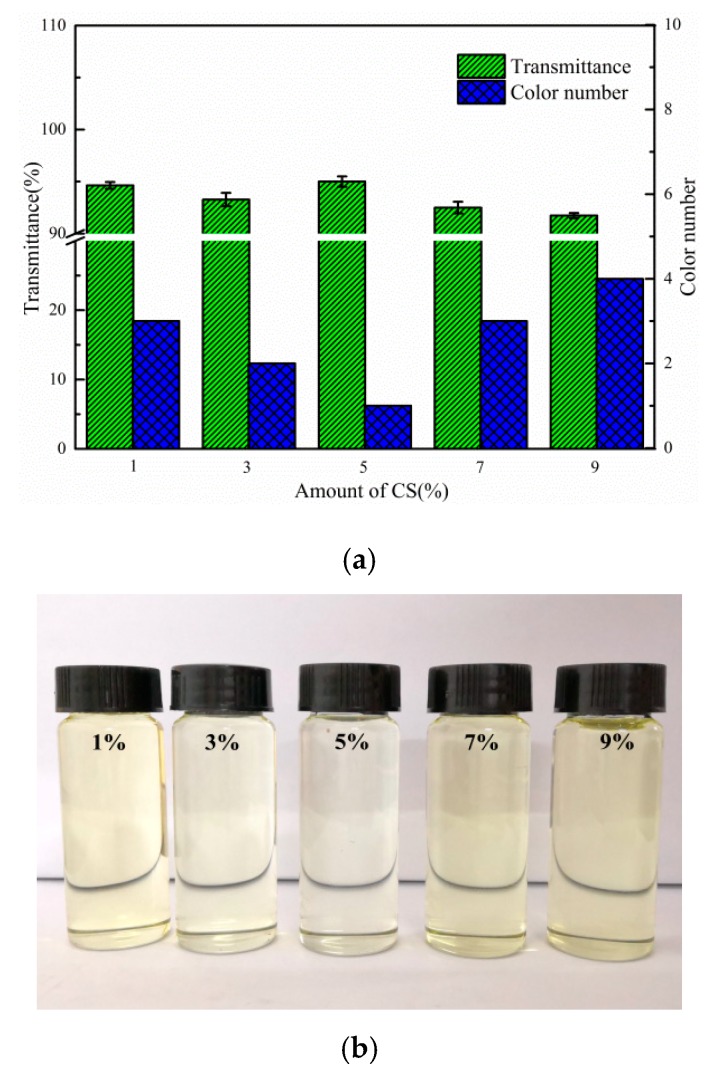
(**a**) Effect of dosage of CS on the transmittance and colour number of the oil (4% of C/BT-300 adsorbent at 50 °C for 2 h). (**b**) Effect of dosage of CS on the appearance of the oil (4% of C/BT-300 adsorbent at 50 °C for 2 h).

Figure 8a,b show the effect of CS dosage on the bleaching capacity of composite adsorbent. With the increase of CS addition, the colour of the transformer oil first decreased, then increased, and it reached a minimum at No. 1 (colour number) when the dosage of CS was 5%. This decrease in colour of the transformer oil could be attributed to the active adsorptive sites formed by carbonised product of CS and BT, which could adsorb colouring matter or gum from the transformer oil, thus increasing the transmittance of the transformer oil and decreasing its colour number. The increasing colour of the transformer oil may be due to the richer carbon content of the coating on the surface of the BT, thus leading to occupation of active sites on the surface of the adsorbent or some channels facilitating the adsorption of colouring matter becoming blocked. Therefore, based on the data set obtained, it could be considered that the optimal CS content was 5%.

### 3.8. Adsorption Experiment

#### 3.8.1. Effect of Adsorbent Content

**Figure 9:**
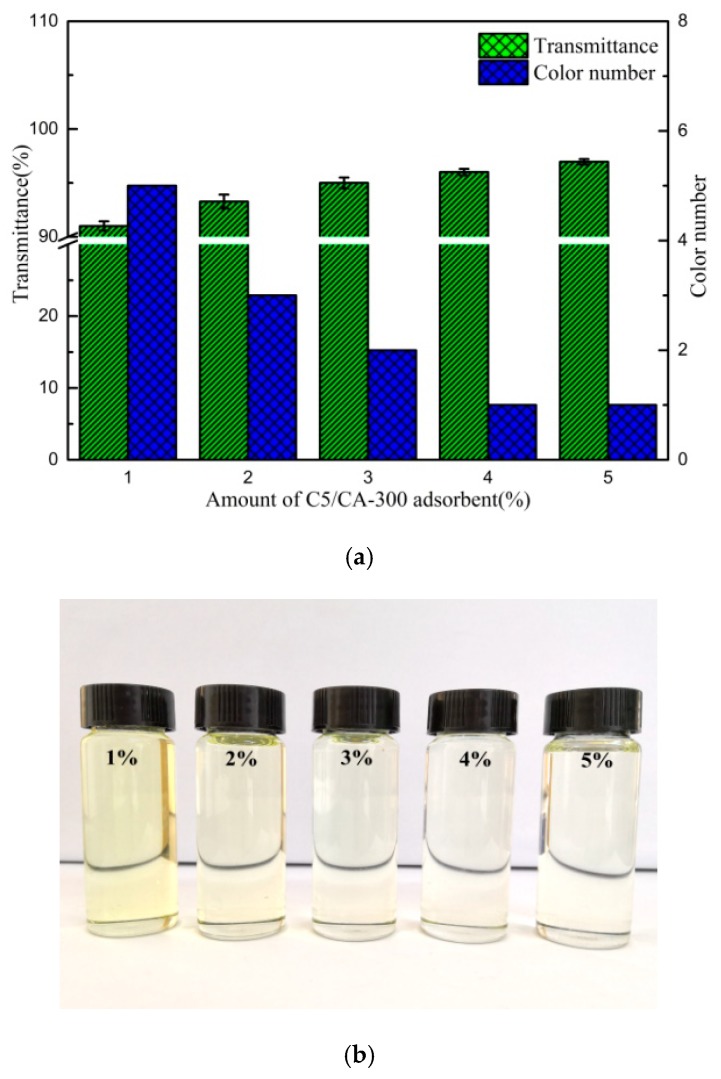
(**a**)Effect of C5/BT-300 content on the transmittance and colour number of oil (50 °C for 2 h). (**b**) Effect of C5/BT-300 content on the appearance of oil (50 °C for 2 h).

Adsorption test data from different contents of C5/BT-300 adsorbent are shown in Figure 9a,b: the initial transmittance and colour number of the transformer oil were 70.1% and no. 10 (Figure 12). As can be seen, the colour number of transformer oil significantly decreased and the transmittance increased with the increased amount of C5/BT-300 adsorbent. This could be attributed to the fact that, when the dosage of adsorbent was low, the adsorbent could not provide sufficient active adsorptive sites to absorb the colouring matters from the transformer oil, however, it was interesting to find that the transformer oil colour number was unchanged and the transmittance increased slightly (from 96% to 96.9%) when the dosage of C5/BT-300 adsorbent increased from 4% to 5%, which could be due to 4% of the adsorbent being sufficient to provide enough active adsorptive sites to absorb the colouring matter from the transformer oil. Consequently, based on the data set obtained, it was considered that the optimal content of C5/BT-300 adsorbent in transformer oil was 4%.

#### 3.8.2. Effect of Adsorption Temperature

**Figure 10:**
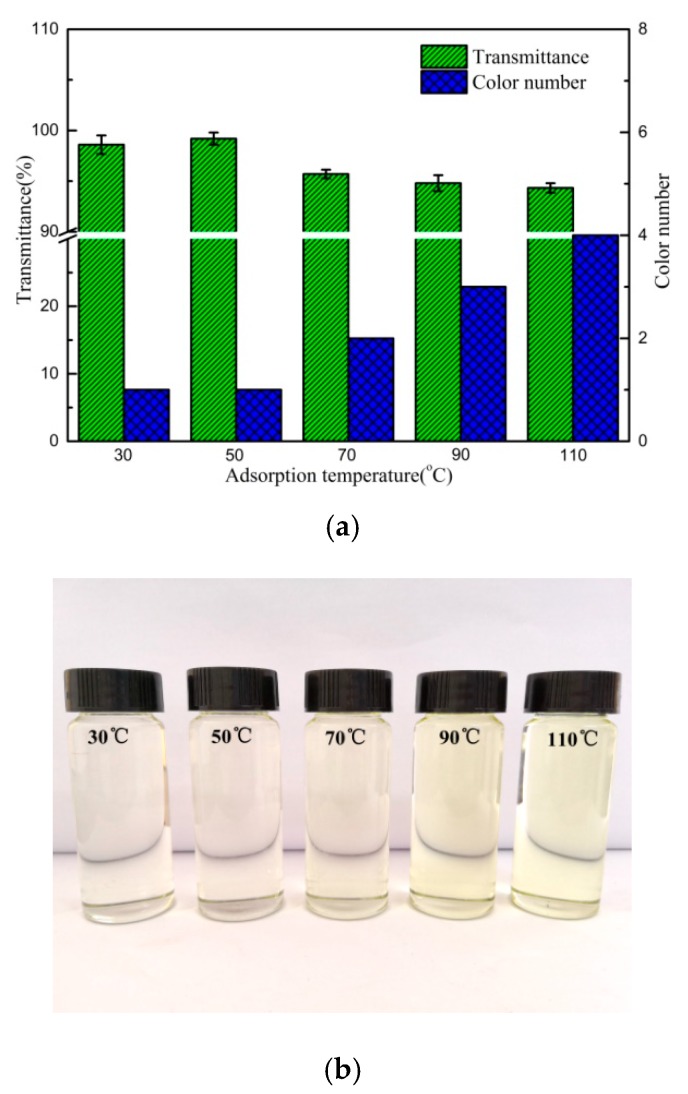
(**a**) Effect of adsorption temperature on the transmittance and colour number of the oil (4% of C5/BT-300 adsorbent for 2 h). (**b**) Effect of adsorption temperature on the appearance of the oil (4% of C5/BT-300 adsorbent for 2 h).

Figure 10a,b show the effect of adsorption temperature on the bleaching capacity of C5/BT-300 adsorbent in the transformer oil. It was found that the bleaching capacity of C5/BT-300 adsorbent was the best at 50 °C but decreased at higher temperatures. When the temperature was 50 °C, the transmittance of the oil reached a maximum of 99.2%, and the colour number also decreased from no. 10 to no. 1, which could be due to the accelerated movement of colouring matter to the boundary of the adsorbent that then improved the effective adsorption rate between the adsorbent and colouring matter. Besides, the viscosity of the oil could be decreased at the appropriate temperature, however, the results suggested that a higher temperature was not beneficial to absorption of colouring matter from the transformer oil, which was attributed to the high temperature promoting desorption of colouring matter and adsorbent. Additionally, the high temperature could also promote chemical reactions between the colouring matter and the transformer oil, thus leading the colour to deepen, effectively aging the oil.

#### 3.8.3. Effect of Adsorption Time

**Figure 11:**
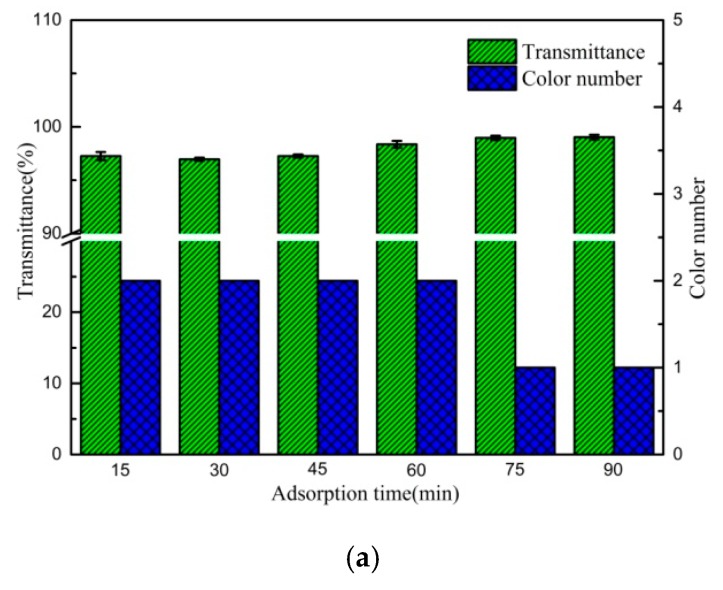

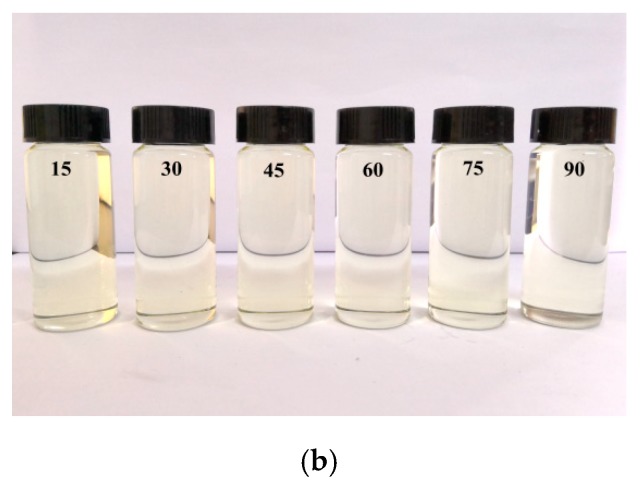
(**a**) Effect of adsorption time on the transmittance and colour number of the oil (4% of C5/BT-300 adsorbent at 50 °C). (**b**) Effect of adsorption time on the appearance of the oil (4% of C5/BT-300 adsorbent at 50 °C).

Results of bleaching tests at different adsorption times were present in Figure 11a,b. Higher adsorption rates were observed at the beginning. When the adsorption time was 15 min, the colour number of the transformer oil decreased from no. 10 to no. 2 and the transmittance increased from 70.1% to 97.3%; however, with further increases in adsorption time, the transmittance increased slightly and the colour number only decreased from no. 2 to no. 1, suggesting that the adsorption equilibrium had been reached, the concentration difference between colouring matter and active sites of the adsorbent may affect the penetration of colouring matter from the oil into/onto the adsorbent surface.

#### 3.8.4. Effect of Different Adsorbents

**Figure 12:**
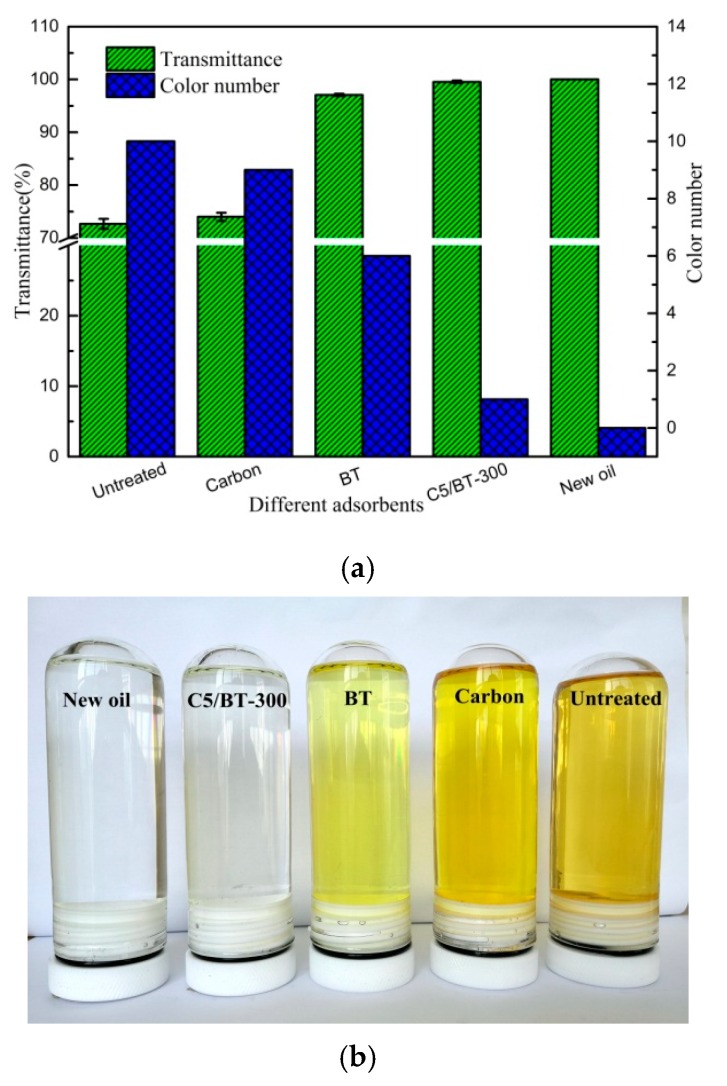
(**a**) Effects of different adsorbents on the transmittance and colour number of the oil (4% of adsorbent at 50 °C for 75 min). (**b**) Effects of different adsorbents on the appearance of the oil (4% of adsorbent at 50 °C for 75 min).

To compare the bleaching capacity of different adsorbents in transformer oil, the most common adsorbents were tested: Figure 12a,b showed the carbon, BT, and C5/BT-300 adsorbent bleaching capacities on transformer oil. The initial transmittance and colour number of the transformer oil were 70.1% and no. 10. During loading with carbon adsorbent, the transmittance and colour number of the oil remained 74% and no. 9, respectively, which indicated that neat carbon cannot reduce the colour of transformer oil effectively. When neat BT was used as the adsorbent, the transmittance of the transformer oil was significantly increased, and colour number also decreased from no. 10 to no. 6, but the oil remained a primrose-yellow colour, however, it was found that the oil which was absorbed by C5/BT-300 adsorbent appeared almost colourless: the transmittance and colour number of the oil could reach 99.5% and no. 1, respectively. This indicated that the C5/BT-300 adsorbent had the best bleaching capacity among those adsorbents tested.

### 3.9. Electrical Properties

The electrical properties of the transformer oil absorbed on different adsorbents are listed in Table 3: before adsorption, the close flash point, breakdown voltage and dielectric dissipation factor of the deteriorating transformer oil were 140 °C, 30.5 kV and 0.32, respectively. During loading with carbon adsorbent, the close flash point, breakdown voltage, and dielectric dissipation factor of the oil were not changed to any significant extent, which indicated that the carbon had no effect on the electrical properties of the transformer oil. When the BT was used as an adsorbent in the transformer oil, these electrical properties have been significantly improved. It was noticeable that after treatment by the C5/CA-300 adsorbent, the close flash point and breakdown voltage of the oil showed the maximum rate of increase, and the dielectric dissipation factor showed the maximum rate of decrease. These results indicated that the adsorption treatment of C5/BT-300 adsorbent was effective, and could improve the electrical properties of the transformer oil.

## 4. Conclusions

Novel C/BT composite adsorbents were prepared by CS and BT through mixing, drying, and calcining and then used for bleaching deteriorating transformer oil. The results showed that excellent bleaching properties of the adsorbent could be achieved given the appropriate proportions of CS and BT and the optimal calcination temperature. FTIR and XRD indicated that there was no chemical interaction between the two components. TGA suggested that the calcination temperature should not exceed 300 °C. SEM, EDX and TEM showed that CS was transformed to carbon and covered the surface of the BT. The adsorption experiment data demonstrated that the C5/BT-300 adsorbent has the highest bleaching capacity compared to carbon and active BT. Electrical experiments involving the deteriorating transformer oil showed that the close flash point and breakdown voltage increased and dielectric dissipation factor decreased with the bleaching process.

## Figures and Tables

**Table 1:** The condition of the calcined samples.

Samples	BT (g)	CS (%)	HCl (%)	Calcination Temperature (°C)
BT-300	15	0	4	300
C5/BT-200	15	5	4	200
C5/BT-250	15	5	4	250
C5/BT-300	15	5	4	300
C5/BT-350	15	5	4	350
C5/BT-400	15	5	4	400
C1/BT-300	15	1	4	300
C3/BT-300	15	3	4	300
C7/BT-300	15	7	4	300
C9/BT-300	15	9	4	300

**Table 2:** Values of specific surface areas (SBET), micropore volume (Vmicro) and desorption average pore width (PW).

Sample	*S*_BET_ (m^2^/g)	*V*_micro_ (cm^3^/g)	PW (nm)
BT-300	141.15	0.195	5.66
CS-300	9.85	0.009	4.77
C5/BT-200	117.44	0.173	5.91
C5/BT-250	114.75	0.174	6.05
C5/BT-300	109.73	0.177	6.10
C5/BT-350	107.25	0.179	6.07
C5/BT-400	106.32	0.180	6.06
C1/BT-300	133.56	0.187	5.87
C3/BT-300	124.87	0.181	5.94
C7/BT-300	108.34	0.173	6.10
C9/BT-300	96.65	0.168	6.14

**Table 3:** Electrical properties of transformer oil.

Treatment	Close Flash Point (°C)	Breakdown Voltage (kV)	Dielectric Dissipation Factor
Untreated	140 ± 3.3	30.5 ± 4.3	0.32 ± 0.17
Carbon	142 ± 2.4	31.7 ± 3.4	0.34 ± 0.09
BT	151 ± 3.8	38.9 ± 2.9	0.26 ± 0.13
C5/BT-300	160 ± 4.5	42.2 ± 4.1	0.19 ± 0.15
New oil (35 kV)	≥135	≥40	≤0.010

## Abbreviations

CS	Chitosan
BT	Bentonite
C	Carbon
